# Individual changes in neurocognitive functioning and health-related quality of life in patients with brain oligometastases treated with stereotactic radiotherapy

**DOI:** 10.1007/s11060-018-2868-7

**Published:** 2018-04-16

**Authors:** Pim B. van der Meer, Esther J. J. Habets, Ruud G. Wiggenraad, Antoinette Verbeek-de Kanter, Geert J. Lycklama à Nijeholt, Hanneke Zwinkels, Martin Klein, Linda Dirven, Martin J. B. Taphoorn

**Affiliations:** 1 Department of Neurology, Leiden University Medical Center, PO BOX 9600, 2300 RC Leiden, The Netherlands; 2 Department of Neurology, Haaglanden Medical Center, The Hague, The Netherlands; 3 Department of Radiotherapy, Haaglanden Medical Center, The Hague, The Netherlands; 4 Department of Radiology, Haaglanden Medical Center, The Hague, The Netherlands; 5 Brain Tumor Center Amsterdam, Amsterdam, The Netherlands

**Keywords:** Health-related quality of life, Neurocognitive functioning, Brain metastases, Stereotactic radiotherapy

## Abstract

**Background:**

Recently, it has been shown that at group level, patients with limited brain metastases treated with stereotactic radiotherapy (SRT) maintain their pre-treatment levels of neurocognitive functioning (NCF) and health-related quality of life (HRQoL). The aim of this study was to evaluate NCF and HRQoL changes over time at the individual patient level.

**Methods:**

NCF (seven domains assessed with a standardized test battery) and HRQoL (eight predetermined scales assessed with the EORTC QLQ-C30 and BN20 questionnaires) were measured prior to SRT and at 3 and/or 6 months follow-up. Changes in NCF and HRQoL were evaluated at (1) a domain/scale level and (2) patient level.

**Results:**

A total of 55 patients were examined, of which the majority showed stable NCF 3 months after SRT, on both the domain level (78–100% of patients) and patient level (67% of patients). This was different for HRQoL, where deterioration in the different scales was observed in 12–61% of patients, stable scores in 20–71%, and improvement in 16–40%, 3 months after SRT. At patient level, most patients (64%) showed both improvement and deterioration in different HRQoL scales. Results were similar between 3 and 6 months after SRT.

**Conclusion:**

In line with results at group level, most brain oligometastases patients with ≥ 6 months follow-up and treated with SRT maintained their pre-treatment level of NCF during this period. By contrast, changes in HRQoL scores differed considerably at domain and patient level, despite stable HRQoL scores at group level.

**Electronic supplementary material:**

The online version of this article (10.1007/s11060-018-2868-7) contains supplementary material, which is available to authorized users.

## Introduction

Brain metastases are a common manifestation of systemic cancer, with an estimated 9–45% of cancer patients developing brain metastases [1, 2]. The increasing incidence of brain metastases is most likely attributable to an aging population, the availability of improved imaging to detect smaller lesions, and better treatment modalities for systemic cancer which prolong life, thereby increasing the risk of dissemination of the systemic cancer to the brain [3, 4]. Although a subgroup of patients experiences longer survival [5], brain metastases are still incurable for most patients and the focus of treatment is mainly palliative [6]. Neurocognitive deficits and a reduced health-related quality of life (HRQoL) are often observed in patients with brain metastases and may be caused by the primary tumor, presence of (brain) metastases themselves, anti-tumor treatment, or supportive medication [4, 7–10]. Although survival is an important treatment endpoint for these patients, maintenance or improvement of neurocognitive functioning (NCF) and HRQoL during the course of the disease are at least as important [11, 12].

Whole-brain radiotherapy (WBRT) has been the standard of care in the past decades, but use of stereotactic radiotherapy (SRT) as an addition to, or as an alternative for, WBRT have increased considerably in recent years [13]. The main component of SRT is precise delivery of focal high dose radiation to a discrete target volume in 1–5 sessions, while minimizing irradiation of surrounding normal tissue [8, 14]. This treatment is particularly useful for patients presenting with limited brain metastases [15], which is the largest subgroup of patients, considering that 70% of the patients have three or fewer metastases [16]. SRT alone is associated with better NCF and HRQoL, while overall survival (OS) is comparable with WBRT alone or a combination of WBRT and SRT [5, 17, 18]. In contrast, SRT alone carries a risk of intracranial recurrences and patients treated with SRT undergo salvage treatment significantly more often compared with patients receiving both WBRT and SRT [19]. These salvage treatments may increase the risk of neurologic deficits and radionecrosis [4, 20].

Habets et al. [21] evaluated NCF and HRQoL prospectively in patients treated with SRT alone for 1–3 brain metastases and found that at group level, NCF and HRQoL remained relatively stable during 6 months from initial treatment, with the exception of physical functioning and fatigue, which worsened over time [21]. Although at group level patients maintained their pre-treatment levels of NCF and HRQoL to a large extent, this may not hold true for all individual patients. Since maintaining or improving NCF and HRQoL is important for all patients treated with SRT, we sought to evaluate changes over time in NCF and HRQoL at the individual patient level.

## Materials and methods

### Study population

Patients were eligible if they were ≥ 18 years; had ≤ 3 newly diagnosed brain metastases (maximum diameter of 4 cm); and were scheduled to undergo SRT, performed on an out-patient base with a dedicated Linac (Novalis; BrainLABAG, Helmstetten, Germany), construction year 2003. Recruitment of patients took place between January 2009 and February 2012. Exclusion criteria were: prior treatment for metastatic brain tumors; insufficient mastery of the Dutch language; and Karnofsky Performance Status (KPS) score < 70. The medical ethics committee of the institution approved the protocol. All patients provided written informed consent.

### Procedures

The gross tumor volume (GTV) was contoured on a contrast-enhanced T1-weighted MRI. Planning target volume (PTV) was created by adding a 2-mm margin by 3D expansion to the clinical target volume (CTV), which was equal to the GTV. SRT treatment consisted of 21 Gy (PTV < 8 cm^3^) or 18 Gy (PTV 8–13 cm^3^) in a single fraction or 24 Gy (PTV > 13 cm^3^ and metastases near the brainstem) in three fractions of 8 Gy.

The baseline evaluation of NCF and HRQoL was conducted in the week preceding SRT. Follow-up assessments took place 3 and 6 months after SRT. Patients’ charts were examined to extract sociodemographic data and clinical variables, including primary tumor, treatment status and medication use. At all time points, MRI scans were made and the status of the primary disease and the use of medication were monitored. If patients showed intracranial progression during follow-up and underwent renewed SRT, provided the number of metastases was ≤ 3, these patients remained in the study. Patients with intracranial progression who transitioned to WBRT were excluded from further assessment. Patients were included in the statistical analysis if they complied for assessment on NCF and/or HRQoL on at least baseline and 3 months, or 3 and 6 months.

### Study instruments

#### Neurocognitive functioning

NCF was assessed with a standardized battery of validated neurocognitive tests found to be clinically relevant in brain tumor patients (Supplementary Table 1) [22–29]. Individual test scores were combined in seven neurocognitive domain scores: verbal memory, visual memory, attention, executive functioning, working memory, information processing speed and visuoconstruction. Raw individual test scores were converted into standardized z-scores, by using means and standard deviations of individually matched healthy controls regarding age, gender, and education level, for four different domains (verbal memory, attention, executive functioning and information processing speed) [30–32]. Published norms were used, corrected for age and education, for the three other domains (visual memory, working memory and visuoconstruction) [33, 34]. A change in z-score of ≥ 1.5 standard deviation (SD) was considered to be clinically meaningful, in line with previous research in the same population [21].

#### Health-related quality of life

HRQoL was evaluated with two validated self-assessment questionnaires, (1) the for cancer patients developed 30-item generic European Organisation for Research and Treatment of Cancer Quality of Life Questionnaire C30 (EORTC QLQ-C30) and (2) the 20-item brain tumour-specific EORTC QLQ-Brain Cancer Module (QLQ-BN20) [35, 36]. A selection of HRQoL scales has been made, based on previous findings [37], comprising six QLQ-C30 scales (global health status, physical functioning, emotional functioning, role functioning, cognitive functioning, and fatigue) and two BN20 scales (motor dysfunction and communication deficits). Global health status was rated on a 7-point Likert scale, ranging from ‘very poor’ to ‘excellent’; the functioning and symptom scales were rated on a 4-point Likert scale, ranging from ‘not at all’ to ‘very much’. Raw scores were converted linearly into standardized scores ranging from 0 to 100. A higher score on the global health status and the functioning scales indicates better HRQoL, while on symptom-oriented scales a higher score indicates worse HRQoL. Difference or change score ≥ 10 points on any given scale were considered to be clinically meaningful [38].

### Statistical analysis

To assess changes in HRQoL and NCF, differences in scores over time were calculated on (1) a domain/scale level and (2) patient level. Above described cut-off scores were used to determine an improvement, deterioration or stable score. Changes in scores were calculated for two different time periods: baseline-3 months, and 3–6 months. A cluster analysis, using R, was performed to identify whether specific HRQoL scales clustered.

Statistical analyses were performed using SPSS version 23.0. Statistical significance for intergroup differences were tested using the *χ*^2^ test for categorical variables, the Student’s t-tests or Mann–Whitney U-test for two-level continuous variables (depending on the distribution of the data), and the Kruskal–Wallis test for continuous variables with more than two levels. Kaplan–Meier curves were used for analyses of OS, and a log rank test to assess differences in survival. A p-value of < 0.05 was considered statistically significant.

#### Domain/scale level

For both time periods (baseline-3 months, and 3–6 months), patients were assigned to one of three categories: (1) deterioration, (2) stable score or (3) improvement, separately for each neurocognitive domain and HRQoL scale. For NCF, improvement and deterioration were defined as an increase or decrease in score ≥ 1.5 SD, respectively, and stable score as < 1.5 SD change. For HRQoL, improvement and deterioration were defined as ≥ 10 points increase or decrease respectively, and stable score as < 10 points increase or decrease.

#### Patient level

At patient level, patients were categorized into four categories, separately for NCF and HRQoL, applying the same cut-off scores as in the domain/scale level. These four categories were as follows: (1) decline, (2) improvement, (3) both and (4) stable. Decline and improvement were defined as deterioration or increase in NCF/HRQoL on at least one domain/scale respectively, while other domains/scales remained stable. The category ‘both’ included both a decline and improvement, whereas ‘stable’ was defined as no detectable change in any neurocognitive domain or HRQoL scale. Moreover, changes in KPS score, SRT dose received (biologically higher [single fraction 21 or 18 Gy] versus lower dosis [8 Gy in three fractions]), total tumor volume (as a proxy for GTV), intracranial progression and active systemic disease were assessed for the four categories, separately for the two time periods.

## Results

Fifty-five out of the original 97 (57%) patients were eligible for analyses, because they had sufficient data. Baseline sociodemographic and clinical characteristics of the study population are summarized in Table 1. These baseline sociodemographic and clinical characteristics were compared between patients with and without sufficient NCF/HRQoL data. At baseline, patients without sufficient data had more often a lower KPS score (median of 80 [inter quartile range (IQR) = 70–80] vs. 80 [IQR = 80–90]; p = .002) and shorter OS (median of 3.8 months [IQR = 1.6–6.4] vs. 12.0 months [IQR = 8.2–12.0]; p < .001) when compared to patients with NCF/HRQoL data. NCF and HRQoL scores over time in our subpopulation were similar to the results as previously reported in the original study population (data not shown).

**Table 1:** Baseline sociodemographic and clinical characteristics of the patient population

	Patients with NCF/HRQoL data	Patients without NCF/HRQoL data	Original study population
Patients included, no. (%)	55	42	97
Age in years, mean ± SD	63 ± 9	64 ± 12	63 ± 11
Sex, no. (%)			
Male	25 (45%)	21 (50%)	46 (47%)
Female	30 (55%)	21 (50%)	51 (53%)
Educational level^a^, median (IQR)	3 (2–4)	2 (2–4)	2 (2–4)
Brain metastases, no. (%)			
1	21 (38%)	22 (52%)	43 (44%)
2	23 (42%)	8 (19%)	31 (32%)
3	9 (16%)	9 (21%)	18 (19%)
4	2 (4%)	3 (7%)	5 (5%)
Tumor volume by patient (cm^3^)			
Median (range)/(IQR)	7.3 (0.12–63.9)/(3.4–12.8)	10.2 (0.15-32.0)/(3.6–15.9)	7.8 (0.12–63.9)/(3.5–14.2)
Missing	1 (2%)	0 (0%)	1 (1%)
Primary cancer, no. (%)			
Non-small cell lung	27 (49%)	20 (49%)	48 (50%)
Renal cell carcinoma	11 (20%)	1 (2%)	12 (13%)
Melanoma	4 (8%)	5 (12%)	9 (9%)
Colorectal cancer	3 (5%)	6 (15%)	9 (9%)
Breast cancer	3 (5%)	5 (12%)	8 (8%)
Other	7 (13%)	4 (10%)	10 (10%)
Missing	0 (2%)	1 (2%)	1 (1%)
Active systemic disease, no. (%)			
Yes	31 (56%)	21 (50%)	52 (54%)
No	24 (44%)	21 (50%)	45 (46%)
Chemotherapy, no. (%)			
Yes	6 (11%)	6 (14%)	12 (12%)
No	47 (85%)	33 (79%)	80 (82%)
Missing	2 (4%)	3 (7%)	5 (5%)
Extracranial metastases, no. (%)			
Yes	29 (53%)	25 (60%)	54 (56%)
No	25 (45%)	16 (38%)	41 (42%)
Missing	1 (2%)	1 (2%)	2 (2%)
Use of corticosteroids, no. (%)			
Yes	48 (87%)	37 (88%)	85 (88%)
No	4 (7%)	4 (10%)	8 (8%)
Missing	3 (5%)	1 (2%)	4 (4%)
Use of AEDs, no. (%)			
Yes	12 (22%)	9 (21%)	21 (22%)
No	40 (73%)	32 (76%)	72 (74%)
Missing	3 (5%)	1 (2%)	4 (4%)
KPS			
Median (IQR)	80 (80–90)	80 (70–80)	80 (70–90)
KPS ≥ 90, No. (%)	25 (46%)	9 (21%)	34 (35%)
Missing	1 (2%)	0 (0%)	1 (1%)
Survival in months, median (IQR)	12.0 (8.2–12.0)	3.8 (1.6–6.4)	7.7 (3.9–12)

Due to rounding, not all percentages add up to 100%

^a^Level 1–8, *NCF* neurocognitive functioning, *HRQoL* health-related quality of life, *SD* standard deviation, *IQR* interquartile range, *AEDs* antiepileptic drugs, *KPS* Karnofsky performance status

## Patient characteristics

The mean age of the 55 included patients was 63 years (SD = 9) and the primary tumor was most frequently located in the lung (49%). Although the MRI scan showed a fourth metastasis in two patients, these patients received SRT because of the small size (< 0.5 cm^3^) and were therefore also included. The median total tumor volume at baseline was 7.3 cm^3^ (IQR = 3.4–12.8) and the 1-year survival rate was 48%, with all patients still alive after 3 months and 87% after 6 months from initial SRT.

## Compliance

During follow-up, compliance dropped from 91% at baseline (n = 50) to 69% at 3 and 56% at 6 months for NCF, and from 98% at baseline (n = 54) to 93% at 3 and 85% at 6 months for HRQoL assessments (Supplementary Table 2). Patients had several reasons for non-compliance, such as progression of disease or the assessment being too demanding.

## Neurocognitive functioning

Prior to SRT, half of the patients with neurocognitive data (25/50, 50%) showed impairments in at least one neurocognitive domain, of which verbal memory was most frequently affected (10/33, 30%).

### Domain level

**Fig. 1:**
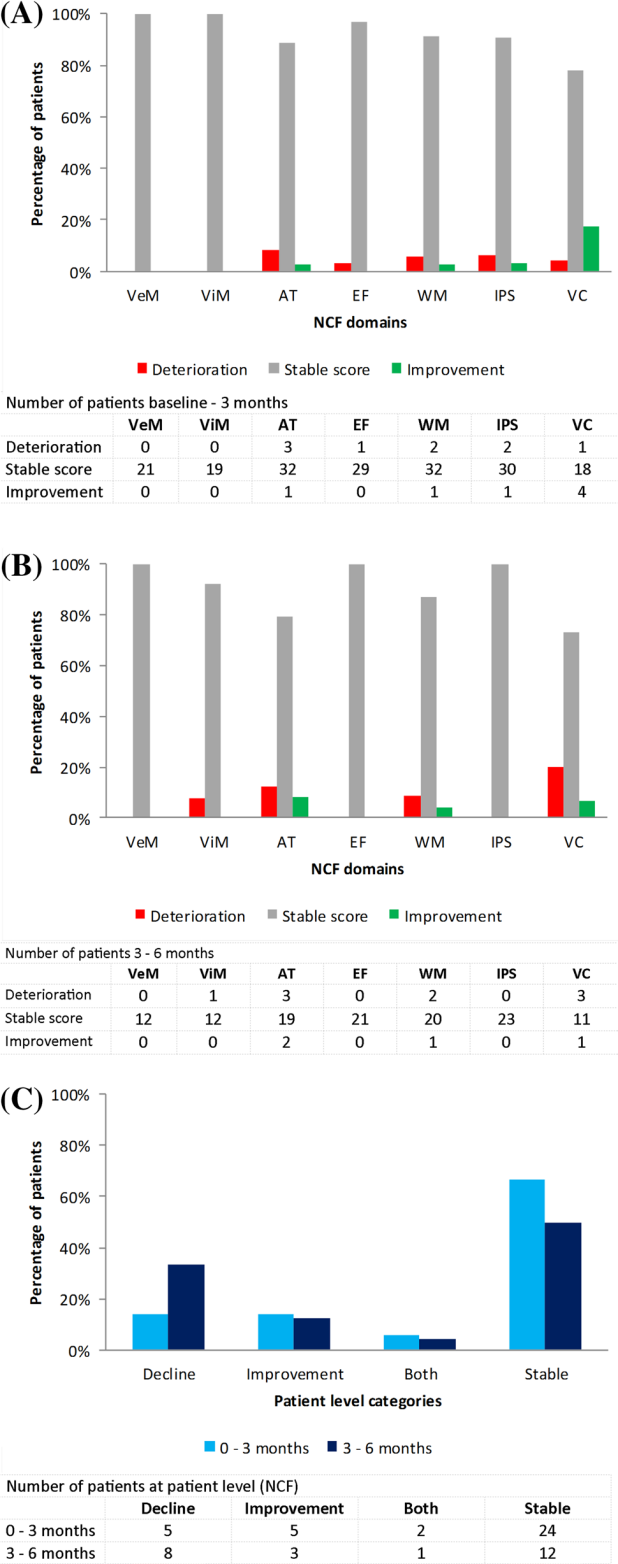
Changes in neurocognitive functioning (NCF) scores calculated from **a** baseline-3 months and **b** 3–6 months at domain level and **c** patient level. *VeM* verbal memory; *ViM* visual memory; *AT* attention; *EF* executive functioning; *WM* working memory; *IPS* information processing speed; *VC* visuoconstruction

Three months after initial SRT, deterioration in the different neurocognitive domains was observed in 5/7 domains (3–8% of patients), while in two domains (verbal memory and visual memory) none of the patients showed deterioration (Fig. 1a). A stable score was observed in all domains (78–100% of patients), most frequently in verbal memory and visual memory. Improvement was found in 4/7 domains (3–17% of patients) and was most profound for visuoconstruction. Similar results were observed between 3 and 6 months after initial SRT [deterioration in 4/7 domains, 8–20% of patients; stable score in all domains, 73–100% of patients; and improvement in 3/7 domains, 4–8% of patients (Fig. 1b)]. Post-hoc analysis using a less stringent cut-off, a change in z-score of ≥ 1.0 SD, revealed similar results (Supplementary Fig. 1a; Supplementary Fig. 1b).

### Patient level

Three months after initial SRT, 14% of patients showed a decline in NCF, another 14% an improvement, 6% both a decline and an improvement, while 67% had stable NCF (Fig. 1c). The period covering 3–6 months after initial SRT revealed similar results (decline 33%; improvement 13%; both 4%; and stable 50%). When using the ≥ 1.0 SD cut-off, scores differed from the original results, but still most patients remained stable or improved (Supplementary Fig. 1c).

Changes in KPS scores (Supplementary Table 3), SRT dose received, total tumor volume, intracranial progression or active systemic disease in individual patients did not differ significantly between the four different categories from baseline to 3 months or from 3 to 6 months after initial SRT.

## Health-related quality of life

Prior to SRT, the vast majority (48/54, 89%) of patients showed a clinically relevant and statistically significant impairment in at least one of the six QLQ-C30 scales when compared to the general population (no reference data available for the QLQ-BN20 scores) [39], of which physical functioning was most frequently affected (31/54, 57%).

### Scale level

**Fig. 2:**
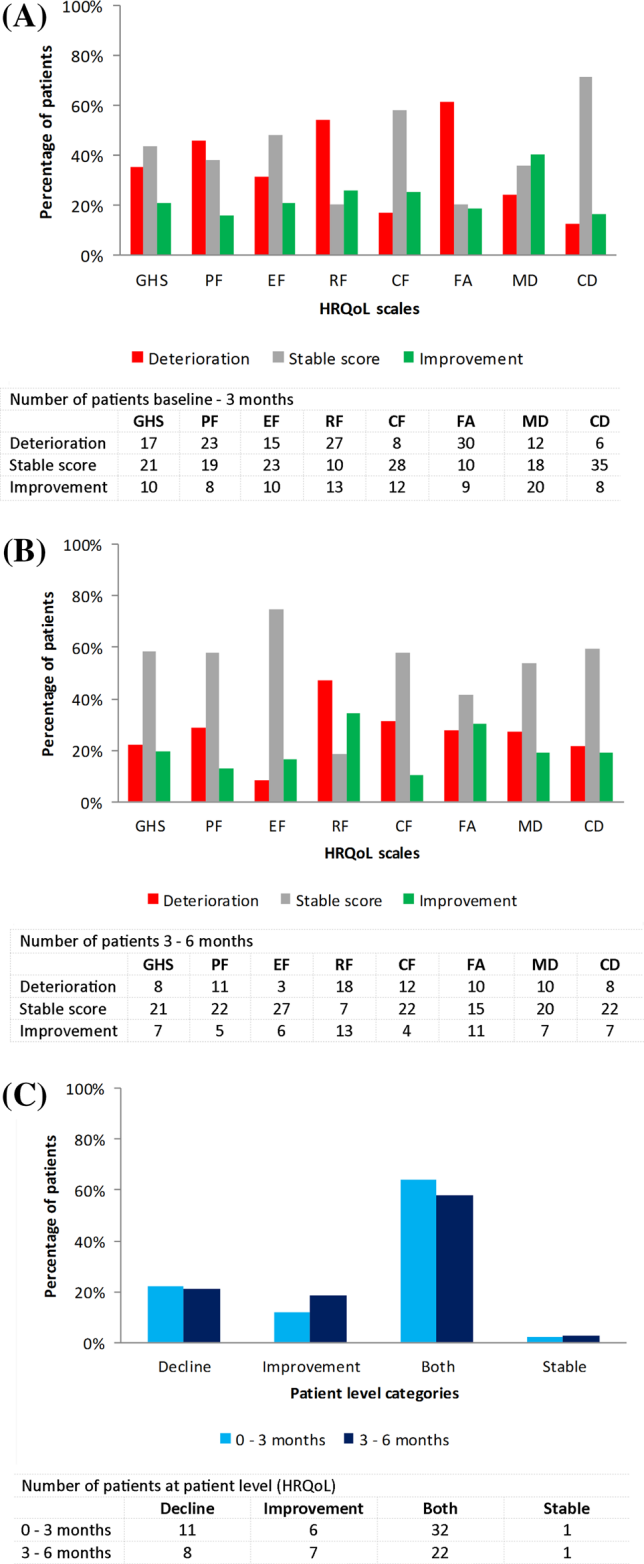
Changes in health-related quality of life (HRQoL) scores calculated from **a** baseline-3 months and **b** 3–6 months at domain level and **c** patient level. *GHS* global health status; *PF* physical functioning; *EF* emotional functioning; *RF* role functioning; *CF* cognitive functioning; *FA* fatigue; *MD* motor dysfunction; *CD* communication deficits

Three months after initial SRT, a decline in all eight different HRQoL scales was observed in 12–61% of patients, most often in fatigue (Fig. 2a). 20–71% of patients had stable scores and an improvement was shown in 16–40% of patients most frequently in communication deficit and motor dysfunction respectively. Comparable percentages were found between 3 and 6 months from initial SRT [deterioration 8–47% of patients; stable score 18–75% of patients; and improvement 11–34% of patients (Fig. 2b)].

### Patient level

A decline in HRQoL in the first 3 months was observed in 22% of patients, an improvement in 12%, both worsening and improvement in 64%, while only 2% had a stable score (Fig. 2c).

Percentages were comparable 6 months after initial SRT (decline 21%; improvement 18%; both 58%; and stable 3%). Changes in KPS scores in individual patients differed significantly between the four categories from baseline to 3 months (p = .001) and from 3 to 6 months (p = .036) after initial SRT, with patients deteriorating in at least one HRQoL scale (in the ‘both’ and ‘decline’ category) showing most often worsening in performance status (Supplementary Table 3). No statistical significant differences between categories were found for SRT dose received, total tumor volume, intracranial progression or active systemic disease (data not shown).

### Cluster analysis HRQoL

**Fig. 3:**
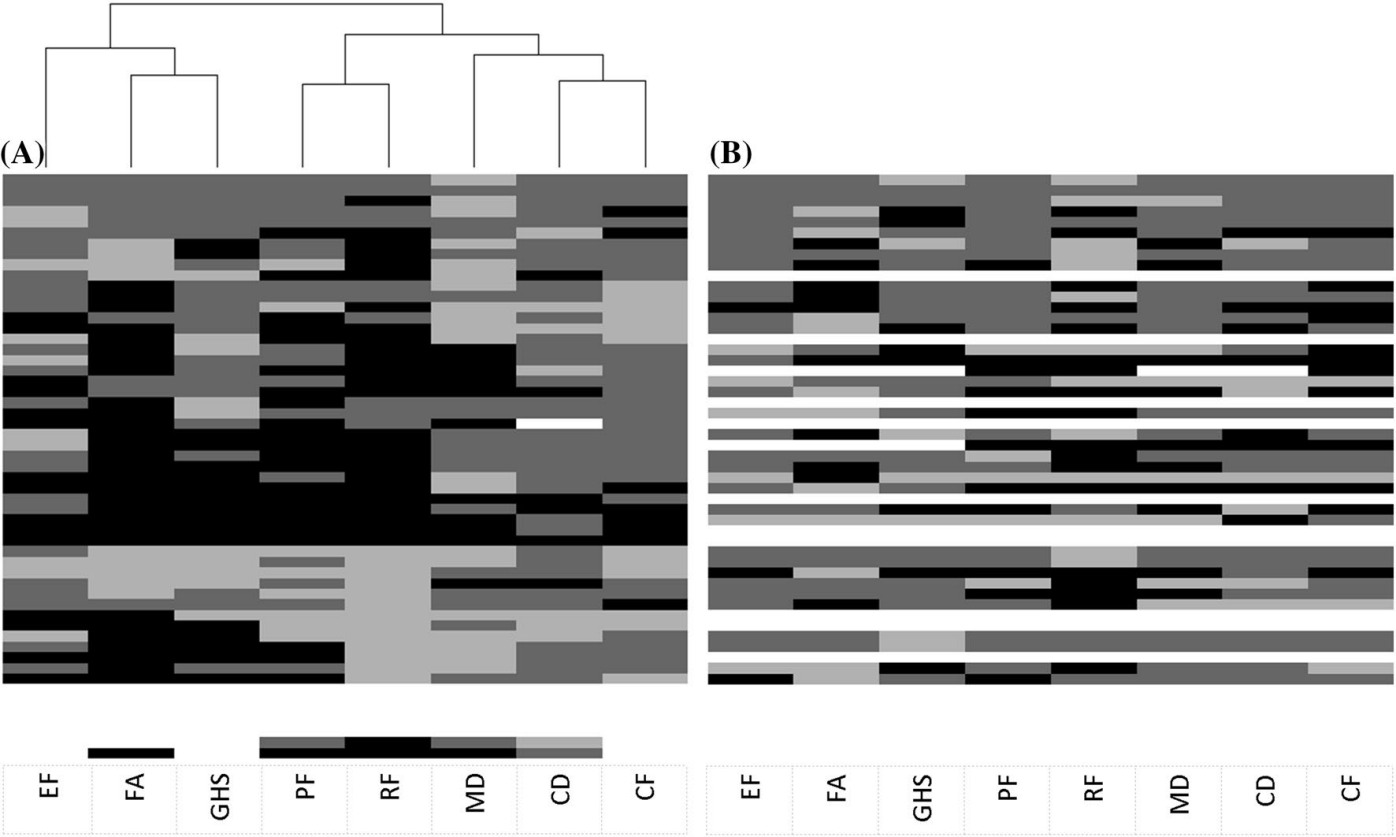
Cluster analysis of differences in health-related-quality of life scores between **a** baseline-3 months and **b** 3–6 months. Black indicates deterioration; dark grey a stable score; light grey improvement; and white a missing value. On the vertical axis all 55 patients included in this study are displayed. **a** Patients are also clustered, but dendrogram is not shown. **b** Patients and HRQoL scales are similarly ordered for comparison. *EF* emotional functioning; *FA* fatigue; *GHS* global health status; *PF* physical functioning; *RF* role functioning; *MD* motor dysfunction; *CD* communication deficits; *CF* cognitive functioning

A heatmap was created to provide insight into changes in HRQoL at patient level (Fig. 3). The most striking pattern is that fatigue, and to a lesser extent emotional functioning, were clustered with global health status, indicating that a change on one scale is likely to be accompanied by a similar change on the other (i.e. decline, improve, both or remain stable). In addition, physical and role functioning were clustered, as well as several brain tumor-specific symptoms, these were motor dysfunction, communication deficit and self-perceived cognitive functioning.

## Discussion

The aim of this study was to evaluate changes in NCF and HRQoL at patient level 3 and 6 months after SRT, providing insight in the impact of treatment on the individual patient level. The overall results, in line with results at group level [21] and several other studies in patients with limited brain metastases [19, 40, 41], indicate that most patients with brain metastases treated with SRT maintained their pre-treatment levels of NCF for at least 6 months. Although NCF and HRQoL at the group level showed little variation, this is not necessarily translated into little variation at domain/scale and patient level. Indeed, changes in scores on the different HRQoL scales did vary substantially within patients, and most individual patients showed both a decline and improvement in separate HRQoL scales in the first 6 months after initial SRT. This finding is in contrast with the HRQoL findings at group level, in which patients who deteriorated and improved most likely cancelled each other out. When informing patients about the impact of a certain treatment or monitor their disease status, it is not sufficient to have information at group level only, nor at the scale level. Clinicians should also be aware that the large majority of patients will experience both deterioration and improvement in HRQoL.

An explanation for the relatively unaffected NCF in brain metastases patients may be that our study population represents a highly selected group of patients with good functioning. Indeed, patients in our sample had a higher KPS score and longer survival compared to our patients without sufficient NCF/HRQoL data. This is also supported by the finding that prior to SRT, only 50% of patients had an impairment in at least one neurocognitive domain, which is considerably lower than in previous studies in metastatic brain tumor patients (67–92%) [40, 42]. Particularly for neurocognitive testing, compliance rates decreased substantially over 6 months’ time. Responsible for non-compliance, among other things, were poor neurological or physical functioning and assessment considered too burdensome. Another explanation is the operational definition of objective neurocognitive decline, for which different cut-offs have been suggested [43]. Brown et al. [44], using a ≥ 1.0 SD cut-off score, found considerably higher neurocognitive deterioration rates compared to our study, with most patients showing cognitive deterioration at 3 months after SRT. However, when using a ≥ 1.0 SD cut-off in our study, still the majority of patients showed no cognitive deterioration, meaning a different cut-off does only partially explains the difference in neurocognitive deterioration rates [44]. Taking into account the aforementioned explanations for the relatively unaffected NCF, maintenance of NCF over 6 months’ time might have been overestimated in our biased sample and likely limits generalizability of the results to brain metastases patients with poor functioning.

Although average HRQoL remained stable at group level, except for physical functioning and fatigue, this did not hold true on scale level nor at patient level. On scale level, patients were relatively similarly distributed over the three different categories (deterioration; stable score; and improvement). At patient level, however, the majority of patients showed both deterioration and improvement in different HRQoL scales after radiotherapy, which has been previously reported in patients with brain metastases, but comparison is difficult because the majority of patients received WBRT instead of SRT [45, 46]. Caissie et al. [45] reported that upon follow-up 1 month after radiotherapy significant improvement was seen in several HRQoL scales, including communication deficit [45]. On the contrary, Steinmann et al. [46] reported that upon follow-up 3 months after the start of radiotherapy patients showed a significant and clinically relevant deterioration in several preselected HRQoL scales, including global health status, physical functioning, fatigue, motor dysfunction and communication deficit, while other scales remained unchanged [46]. In our study, the majority of patients showed a clinically relevant deterioration between baseline and 3 months in physical functioning (46%), role functioning (54%) and fatigue (61%), reflecting the findings at group level [21]. Nevertheless, considering the varying trajectories of changes in HRQoL after SRT, an important observation is that the majority of our patients showed both decline and improvement in separate HRQoL scales. An explanation for the varying trajectories of changes is that HRQoL measures vastly different concepts, encompassing physical, emotional, and social components, and that this outcome may be influenced by many factors, including comorbidity, marital status, heterogeneity of the primary tumor, SRT dose, total tumor volume, progression of the extracranial cancer and its corresponding supportive or anti-tumor treatment [47]. Although, SRT dose received, total tumor volume, intracranial progression and active systemic disease did not differ significantly between the four different categories at patient level, this result must be interpreted with caution due to our small sample size. As pointed out by Wilson and Cleary [48] in their model, more distal measures to the disease or the treatment (i.e. global health status and the functioning scales) are not only affected by health status but also by non-medical factors, as opposed to more proximal measures (i.e. symptoms) [48]. NCF is a proximal measure, which is mainly influenced by the presence of brain metastases, or its treatment. Patients who deteriorated on at least one HRQoL scale did most often have decreased performance status, suggesting that especially the patients’ overall functioning influences HRQoL. Moreover, Caissie et al. [49] found that fatigue and emotional functioning were the two strongest predictors of global health status in brain metastases patients, which is similar to the findings of our cluster analysis; deterioration in global health status clusters with increased fatigue and worse emotional functioning, suggesting fatigue may be a target for intervention to improve overall HRQoL [49].

To conclude, in accordance with previous results at group level, this study showed that most patients with brain oligometastases treated with SRT maintained their pre-treatment NCF for at least 6 months. However, changes in scores for the various HRQoL scales differed considerably between and within patients, suggesting that overall functioning is determined by complex underlying mechanisms which should be further analysed.

## Electronic supplementary material

Below is the link to the electronic supplementary material.

Supplementary material 1 (PDF 316 KB)
