# Supplementary material for: Individual changes in neurocognitive functioning and health-related quality of life in patients with brain oligometastases treated with stereotactic radiotherapy
Source: J Neurooncol. 2018 Apr 16;139(2):359–68. doi: 10.1007/s11060-018-2868-7 (PMC6096889; doi:10.1007/s11060-018-2868-7)
Supplement: Supplementary file 1 — Supplementary material 1 (PDF 316 KB) [file 11060_2018_2868_MOESM1_ESM.pdf]

Supplementary material

Individual changes in neurocognitive functioning and health-related quality of life in patients with brain oligometastases treated with stereotactic radiotherapy

Pim B. van der Meer, Esther J.J. Habets, Ruud G. Wiggenraad, Antoinette Verbeek-de Kanter, Geert J. Lycklama à Nijeholt, Hanneke Zwinkels, Martin Klein, Linda Dirven, Martin J.B Taphoorn

Content:

Supplementary Table 1 Neurocognitive domain and the corresponding neurocognitive test(s)

Supplementary Table 2 Compliance with NCF and HRQoL assessments

Supplementary Table 3 Changes in KPS scores from baseline - 3 months and 3 - 6 months, with respect to changes in NCF and HRQoL at patient level

Supplementary Fig. 1 Changes in neurocognitive functioning (NCF) scores, using a change in z-score of ≥1.0 SD, at domain level calculated from **a** baseline - 3 months and **b** 3 - 6 months, and **c** patient level

Supplementary Table 1 Neurocognitive domain and the corresponding neurocognitive test(s)

Neurocognitive domain	Neurocognitive test(s)
Verbal memory	Rey Auditory Verbal Learning Test (immediate recall, delayed recall and delayed recognition)[26]
Visual memory	Rey Complex Figure Test (copying and delayed recall)[31]
Attention	Stroop Color-Word Test[24]
	Digit Span forward and backward, a subtest of Wechsler Adult Intelligence Scale III[27]
Executive functioning	Concept Shifting Test[28]
	Word fluency (category and letter fluency)[24]
	Key-search test, a subtest of the behavioural Assessment of the Dysexecutive Syndrome[29]
Working memory	Digit Span forward and backward, a subtest of Wechsler Adult Intelligence Scale III[27]
Information processing speed	Stroop Color-Word Test[24]
	Letter Digit Substitution Test[24]
Visuoconstruction	Rey Complex Figure Test (copying and delayed recall)[31]

Supplementary Table 2 Compliance with NCF and HRQoL assessments

	No. of Forms/ Assessments Expected[a]	No. of forms completed/ Assessments performed	
		NCF	HRQoL
Baseline	55	50 (91%)	54 (98%)
3 months	55	38 (69%)	51 (93%)
6 months	48	27 (56%)	41 (85%)
Baseline, 3 and 6 months	48	23 (48%)	37 (77%)

[a]Based on alive participating patients, NCF = neurocognitive functioning, HRQoL = health-related quality of life

Supplementary Table 3 Changes in KPS scores from baseline - 3 months and 3 - 6 months, with respect to changes in NCF and HRQoL at patient level

	Changes in KPS scores (0-3 months, n=36; 3-6 months, n=24)					
	Deterioration		Stable		Improvement	
	0-3 months	3-6 months	0-3 months	3-6 months	0-3 months	3-6 months
NCF, No. (%)						
Decline	1 (3)	1 (4)	3 (8)	5 (21)	1 (3)	2 (8)
Both	1 (3)	0 (0)	0 (0)	0 (0)	1 (3)	1 (4)
Stable	14 (39)	4 (17)	6 (17)	6 (25)	4 (11)	2 (8)
Improvement,	1 (3)	0 (0)	1 (3)	2 (8)	3 (8)	1 (4)
HRQoL, No. (%)						
Decline	10 (20)	4 (11)	0 (0)	4 (11)	1 (2)	0 (0)
Both	15 (30)	6 (16)	12 (24)	13 (34)	5 (10)	3 (8)
Stable	1 (2)	1 (3)	0 (0)	0 (0)	0 (0)	0 (0)
Improvement	0 (0)	0 (0)	1 (2)	3 (8)	5 (10)	4 (11)

KPS = Karnofsky Performance Status, NCF = neurocognitive functioning, HRQoL = health-related quality of life
Note: Due to rounding, not all percentages add up to 100%

Supplementary Fig. 1 Changes in neurocognitive functioning (NCF) scores, using a change in z-score of ≥1.0 SD, at domain level calculated from **a** baseline - 3 months and **b** 3 - 6 months, and **c** patient level. VeM, verbal memory; ViM, visual memory; AT, attention; EF, executive functioning; WM, working memory; IPS, information processing speed; VC, visuoconstruction

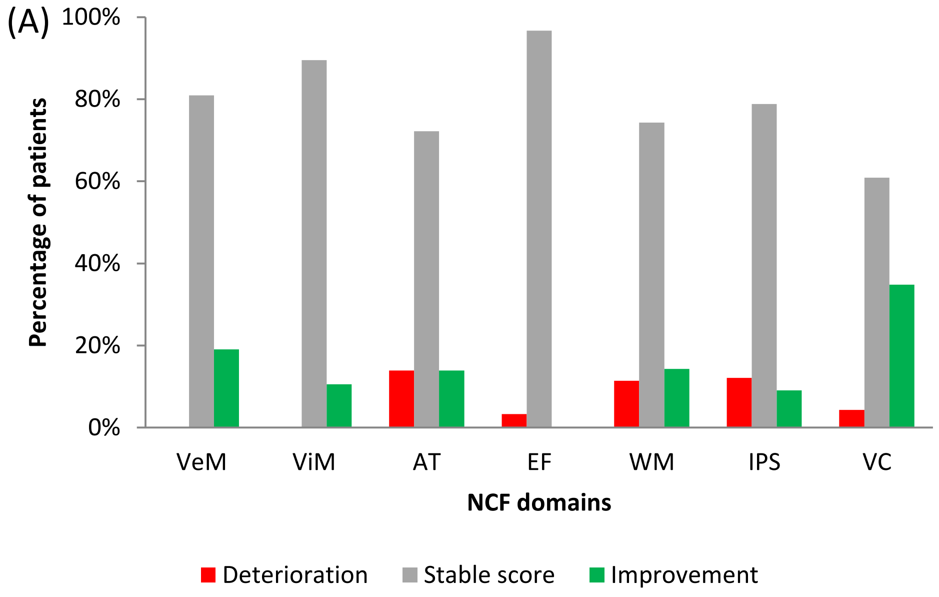

Number of patients at domain level, baseline - 3 months

	**VeM**	**ViM**	**AT**	**EF**	**WM**	**IPS**	**VC**
Deterioration	0	0	5	1	4	4	1
Stable score	17	17	26	29	26	26	14
Improvement	4	2	5	0	5	3	8

(B)

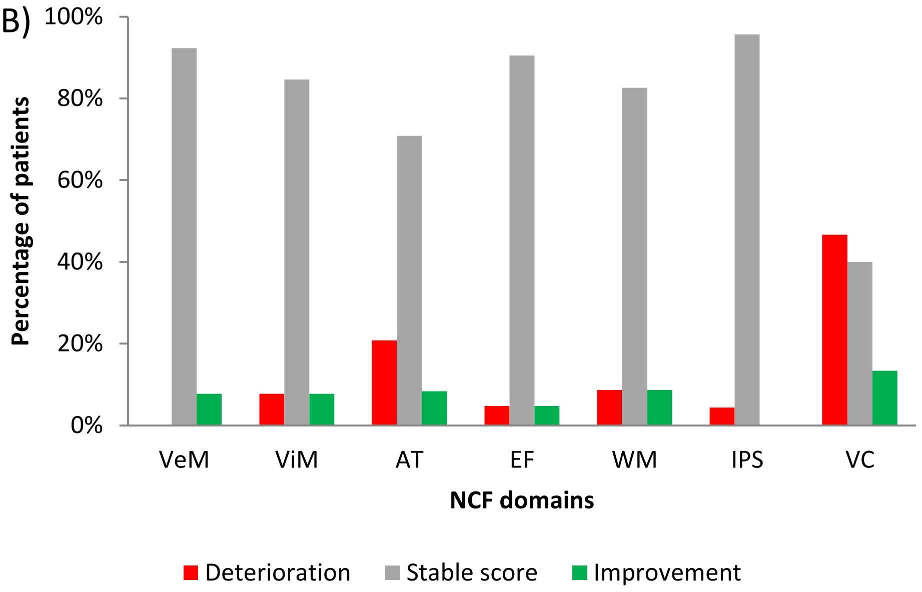

Number of patients at domain level, 3 - 6 months

	**VeM**	**ViM**	**AT**	**EF**	**WM**	**IPS**	**VC**
Deterioration	0	1	5	1	2	1	7
Stable score	12	11	17	19	19	22	6
Improvement	1	1	2	1	2	0	2

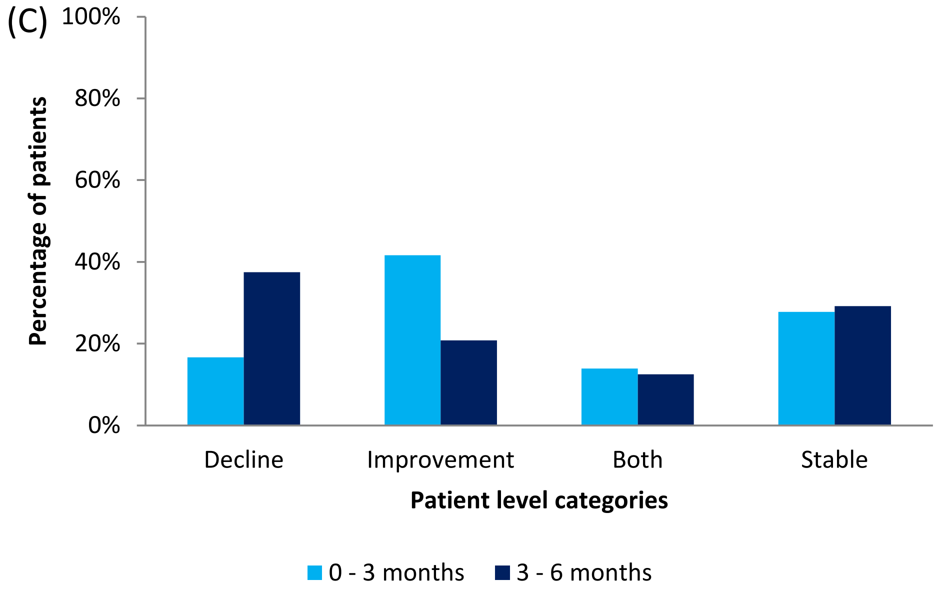

Number of patients at patient level (NCF)

	Decline	Improvement	Both	Stable
0 - 3 months	6	15	5	10
3 - 6 months	9	5	3	7
